# Toward a Robust Security Paradigm for Bluetooth Low Energy-Based Smart Objects in the Internet-of-Things

**DOI:** 10.3390/s17102348

**Published:** 2017-10-14

**Authors:** Shi-Cho Cha, Kuo-Hui Yeh, Jyun-Fu Chen

**Affiliations:** 1 Department of Information Management, National Taiwan University of Science and Technology, Taipei 10607, Taiwan; csc@cs.ntust.edu.tw (S.-C.C.); D9909302@mail.ntust.edu.tw (J.-F.C.); 2 Department of Information Management, National Dong Hwa University, Hualien 97401, Taiwan

**Keywords:** Bluetooth low energy, internet-of-things, privacy, random address, security

## Abstract

Bluetooth Low Energy (BLE) has emerged as one of the most promising technologies to enable the Internet-of-Things (IoT) paradigm. In BLE-based IoT applications, e.g., wearables-oriented service applications, the Bluetooth MAC addresses of devices will be swapped for device pairings. The random address technique is adopted to prevent malicious users from tracking the victim’s devices with stationary Bluetooth MAC addresses and accordingly the device privacy can be preserved. However, there exists a tradeoff between privacy and security in the random address technique. That is, when device pairing is launched and one device cannot actually identify another one with addresses, it provides an opportunity for malicious users to break the system security via impersonation attacks. Hence, using random addresses may lead to higher security risks. In this study, we point out the potential risk of using random address technique and then present critical security requirements for BLE-based IoT applications. To fulfill the claimed requirements, we present a privacy-aware mechanism, which is based on elliptic curve cryptography, for secure communication and access-control among BLE-based IoT objects. Moreover, to ensure the security of smartphone application associated with BLE-based IoT objects, we construct a Smart Contract-based Investigation Report Management framework (SCIRM) which enables smartphone application users to obtain security inspection reports of BLE-based applications of interest with smart contracts.

## 1. Introduction

With the advancement of wireless communications and pervasive computing technologies on smartphones, people can control nearby Internet-of-Things (IoT) devices, e.g., wearable devices or fixed specific-purpose sensors, via their smartphones. Versatile IoT-based service applications have been developed on the smartphone to provide intelligence and convenience to our daily life. Because major smartphone platforms, such as iOS and Android, are equipped with Bluetooth Low Energy (BLE) capabilities and have well-implemented API for BLE communication, more and more IoT devices have adopted BLE technologies to communicate with smartphones. To ensure that users have permissions to access BLE-based IoT devices, the devices usually request users to provide credentials as access tokens for the purpose of authentication and access control. In general, users will request the credentials via an Internet service. In that case, users can obtain credentials with online Internet services, and accordingly the costs for users for installing credentials into their smartphones can be reduced. Alternatively, sellers of the devices will provide physical media, like the PIN code protection envelope provided by banks, to deliver credentials (or key phrases) to users. Nevertheless, there are management issues in protecting the printed out credentials from being stolen by malicious people.

Since privacy has become a critical issue in smartphone applications, current smartphone platforms have started to prohibit smartphone applications from obtaining sensitive information, such as a smartphone’s physical identification, which may result in potential individual privacy disclosure. For example, after Android 6.0 [1], if Android applications invoke the BluetoothAdapter.getAddress() method to get the Bluetooth hardware address of a smartphone, the applications will receive a constant value of 02:00:00:00:00:00 instead of the complete address value. Moreover, to prevent user smartphones from being tracked by malicious adversaries using smartphone Bluetooth Media Access Control (MAC) addresses, a useful technique, called BLE random address, is adopted on smartphones [2,3]. That is, the smartphone will adopt different Bluetooth MAC addresses to communicate with nearby BLE-based devices when the smartphone restarts its Bluetooth function. After a device has been paired with another device with the BLE random address scheme, the device will then transfer the original Bluetooth MAC address and associated identification data to another device. After that, when the device picks up a new Bluetooth MAC address during the next session, the linked device can use the information to resolve the new address into the original one. Note that a device may still give random addresses to paired devices rather than its original address for privacy considerations [3]. However, if a device does not provide its original address to paired devices, the device may need to execute the pairing process again.

**Figure 1:**
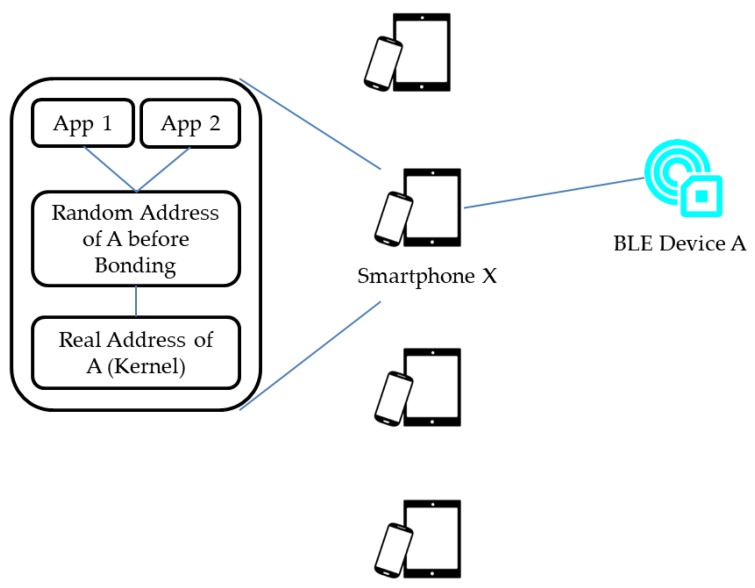
A potential security impact of adopting the random address technique.

Although the random address scheme in BLE can prevent BLE-based IoT devices from being tracked by unauthorized parties, once a smartphone has bonded to a device using the random address mechanism, every application installed on this smartphone can track the device. For example, in Figure 1, suppose that a BLE device A adopts the random address scheme to protect its real Bluetooth MAC address. The smartphone X cannot obtain the real Bluetooth MAC address of the device A. For example, assume there exists a smartphone X with the App 1 installed on the smartphone. The user of smartphone X can initiate the pairing process to establish a pairing relationship between the smartphone X and the device A via the App 1. The smartphone X can accordingly obtain the real Bluetooth MAC address of the device A. In order to prevent the App 1 from obtaining the real address of the device A, the smartphone X stores the latest random address of the device A before pairing and provides the stored address to the App 1. Therefore, the App 1 can identify the device A with the stored address. Comparatively, other smartphones aside from X cannot know how to link received Bluetooth MAC addresses to the device A unless there is only one nearby device. Unfortunately, to ensure the effectiveness of random addresses a BLE-based device may not allow simultaneous pairing connections to the device. Consequently, many BLE applications are designed without pairing. This paves a way for malicious adversary to retrieve sensitive data or to launch a user privacy invading attack. As illustrated in Figure 1, the App 2 can also obtain the stored address to identify the device A without any newly invoked pairing process. In our previous work [4], a security weakness based on the above scenario had been identified in a commercial product sold in Taiwan, i.e., a smart motorcycle called Gogoro Smart Scooter [5]. In general, users can control their Gogoro motorcycles through BLE signals sent from the user’s smartphone. Based on the reverse-engineering analysis on the application associated with Gogoro motorcycles, in the following we present the communication processes of starting a Gogoro motorcycle. Note that the processes occur between Gogoro motorcycles, smartphones and backend servers. 

Each motorcycle has a public Bluetooth address and a secret key stored in its engine control unit. The backend server built by the Gogoro company firstly stores addresses and secret keys of every motorcycle. When a user buys a motorcycle S, the Gogoro company requests the user to provide his/her email and a password as a registration request. Next, the user can install an application on his/her smartphone which will be mainly used for logging into a web server provided by the company. After verifying the user by the provided email and password, the server sends a secret key $K_{S}$ and a Bluetooth address $A_{S}$ associated with the motorcycle S to the application. The motorcycles use a customized pairing process to pair with user smartphones: the motorcycles just store the addresses of their owners’ smartphones. However, because current smartphones usually use random addresses, the backend server of the company generates pseudo-addresses for user smartphones. After successfully authenticating against the backend server, the user U can obtains his/her pseudo-address $A_{U}$ from the backend server and provides the address to his/her motorcycle. When the S wants to use his/her smartphone to control the motorcycle, the smartphone advertises a message with the address of the motorcycle, i.e., $A_{S}$, and his/her pseudo-addresse, i.e., $A_{U}$. After receiving the message, the motorcycle can detect the presence of the user’s smartphone and try to connect to the specific GATT service in the smartphone and send a challenge back to the application. The motorcycle application on the smartphone then retrieves the challenge and generates a response based on the secret key of the motorcycle and the challenge. Finally, the motorcycle judges whether the the response matches the challenge. If the response matches the challenge, the motorcycle executes the previously received command. Nevertheless, the above process has a vulnerability. If malicious people can obtain the pseudo-addresses of motorcycle owners, Bluetooth MAC addresses of motorcycles, and secret keys of the motorcycles, those malicious people can control motorcycles on behalf of motorcycle owners. The pseudo-addresses of smartphones that can control motorcycles and addresses of motorcycle can easily be retrieved because the motorcycle applications advertise the pseudo-addresses of user smartphones and the addresses of motorcycles to notify associated motorcycles to connect to the smartphones. Moreover, based on our analysis, although the application uses SSL to communicate with the Web server of the company, the application does not verify the web server certificate. Therefore, malicious people may intercept the communication between the web server and user applications to obtain secret keys to control associated motorcycles (the company has fixed the issue after being notified by us). Obviously, using pseudo-addresses would not be a good solution to identifying devices using random addresses.

**Figure 2:**
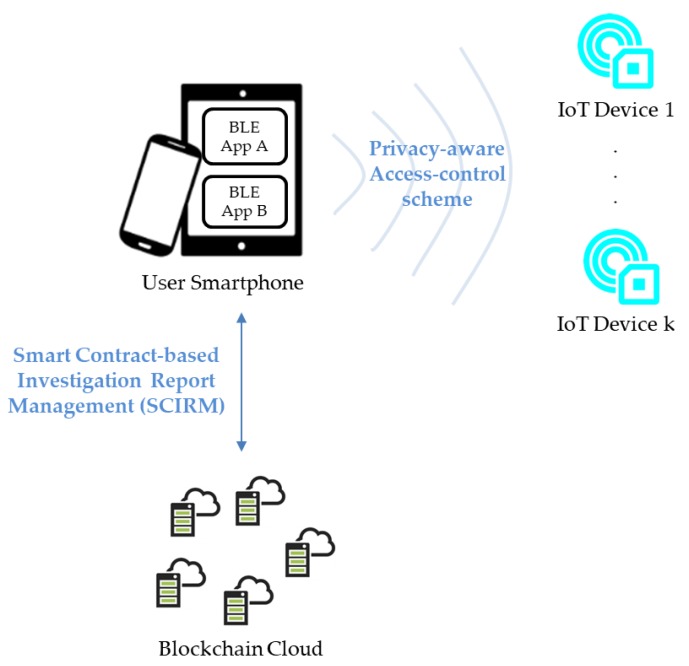
The proposed security paradigm for BLE-based applications associated with IoT devices.

Because the pairing process plays an important role in the BLE-based authentication process for preventing Man-In-the-Middle (MITM) attacks and providing communication security, BLE applications without pairing would increase security risks on the smartphone (and to its owner). Therefore, in this study we first present the generalized requirements BLE-based applications associated with IoT devices. From the viewpoint of IoT devices, to pursue the best tradeoff between privacy and security, we demonstrate a privacy-aware access control scheme for BLE-based IoT devices. Since in our scheme BLE-based IoT applications can constrain an authorized person to use a specific smartphone to access IoT devices without revealing the smartphone’s physical identification information, the proposed method can contribute to alleviate the tension between security and privacy on BLE-based IoT applications. Moreover, from the viewpoint of a smartphone application associated with the IoT objects, we propose a Smart Contract-based Investigation Report Management (SCIRM) framework for the security management of BLE-based applications. The SCIRM enables smartphone application users to obtain security inspection reports of BLE-based applications of interest with smart contracts. Benefiting from blockchain technology, users can obtain historical inspection reports of a BLE-based application and verify the integrity of the reports. In addition, SCIRM utilizes smart contract technology to implement the interfaces so that smart contracts will enforce the related actions automatically. The presented framework can enable users to adopt appropriate countermeasures to potential application security risks as users can obtain up-to-date security information about applications in a timely way. In brief, in this study we introduce a robust security paradigm, as shown in Figure 2, for BLE-based smart objects in the IoT in which two viewpoints on IoT device security and smartphone applications security, respectively, are the focus. The contribution of this manuscript is twofold: one is a privacy-aware access control scheme for secure communication between the smartphone and BLE-based IoT devices and the other one is the SCIRM for the security management of BLE-based applications.

The rest of the paper is organized as follows: Section 2 discusses the related work and Section 3 presents the generalized security requirement of BLE-based applications associated with IoT devices. In Section 4, we introduce a privacy-aware access-control mechanism to fulfill the security criterion we have proposed. After that, the proposed framework, i.e., SCIRM, is presented in Section 5 and, finally, we conclude the paper in Section 6.

## 2. Related Work

In recent years, researchers have paid a great deal of attention to the development of IoT-based applications with potential security and privacy issues. In 2012, Jara et al. [6] designed a knowledge acquisition and management platform relying on IoT network architecture. The platform focused on the management of personal health, and enabled delivery of healthcare services by virtue of its capabilities to predict health anomalies in real-time and offer feedback to patients. The next year, Berhanu et al. [7] presented an adaptive security process for IoT devices in an e-Health environment, and examined the validation through the study of the impact of antenna orientation on energy consumption. The authors investigated the feasibility of adopting lightweight security solutions as part of the ASSET infrastructure [8]. In 2014, Torjusen et al. [9] proposed a solution integrating run-time verification enablers in the feedback adaptation loop of the ASSET system, i.e., an adaptive security framework in an IoT based e-Health environment, and implemented the framework with colored Petri Nets. The run-time enablers produce machine-based formal models of a system’s status and available context at run-time. Moreover, the authors presented requirements for verification at run-time as formal specifications and introduced dynamic context monitoring and adaptation. Recently, Gope and Hwang [10,11] proposed authentication mechanisms for a distributed IoT-based network architecture and applied the proposed techniques to healthcare management. The protocols are suitable for Body Sensor Networks consisting of health care-oriented sensor nodes. To establish secure communication in an efficient way, lightweight cryptography modules, i.e., one-way hash function and bitwise exclusive-or operation, are adopted to support the authentication process. The authors then investigated the security and performance via BAN logics inference and protocol efficiency analysis.

In view of the security issues of the IoT architecture, Yao et al. [12] presented a lightweight multicast communication scheme on a small scale level of IoT applications. The proposed method is based on the fast one-way accumulator technique [13] which provides high usage flexibility when existing and accumulated items in an application are dynamic. The authors then reconstruct a fast one-way accumulator and designed a lightweight multicast authentication mechanism for small scale IoT applications. Based on the evaluation and analysis, this study showed that the proposed multicast authentication scheme can meet the requirements of resource-constraint applications. In 2015, to achieve security protection among ubiquitous things, Ning et al. [14] proposed an aggregated proof- based hierarchical authentication scheme for layered U2IoT architectures. In the presented scheme, anonymous data transmission, mutual authentication and hierarchical access control are achieved via user authorization, aggregated-proof verifications, homomorphism functions and Chebyshev chaotic maps. The authors claimed that their proposed scheme is suitable for the U2IoT architecture. Later, Hernández-Ramos et al. [15] developed several lightweight authentication and authorization schemes, which are compliant with the Architectural Reference Model (ARM) from the EU FP7 IoT-A project, on constrained smart objects. The proposed schemes can be combined with other standard technologies and form security plans for the lifecycle of IoT devices. The same year, Kawamoto et al. [16] implemented a flexible data collection scheme for retrieving ambient information from intelligent sensors/devices as authentication tokens in a location-based authentication system for industrial IoT applications. In the proposed method, a dynamic parameter adjusting mechanism is automatically performed based on system factors and surrounding environment parameters to pursue higher system accuracy. Next, Cirani et al. [17] presented an integration architecture consisting of HTTP/CoAP services and open authorization (OAuth) services for IoT applications with multiple constrained objects which are limited by their computational power. The authors then conducted a performance evaluation via simulations with Contiki OS-based constrained devices to present the feasibilities and practicability of their proposed architecture. More recently, Cha et al. [4] presented an analysis for examining the security impact of adopting random address technique in BLE-based IoT applications. The authors argued that a one-to-one property must be strictly adopted for credentials and device connection. Afterwards, the authors presented a privacy enhancement solution for BLE-based IoT devices. Fawaz et al. [18] examined more than 200 types of BLE-based devices and discovered these devices cannot fulfill the privacy criteria, i.e., the device’s presence may be revealed. Potential threats, such as the revealing of individual sensitive information and even behavior tracking, may thus exist. To conquer the problem, the authors proposed a privacy-aware enhancement system, called BLE-Guardian, which delivers robust privacy protection for users (or environments) equipped with BLE devices. In the proposed system, the users (and administrators) possess the ability to monitor and control those who discover, scan and connect to their devices. Then, Rizzardia et al. [19] added security functionalities to MQTT primitives and accordingly constructed a lightweight data-sharing system. With the support of a policy management scheme, the flow of information in MQTT-enabled IoT systems can be flexibly controlled based on predefined flexible policies. Furthermore, the authors presented the proposed mechanism as an open source feature under an Apache v.2 license.

## 3. The Generalized Requirements

**Figure 3:**
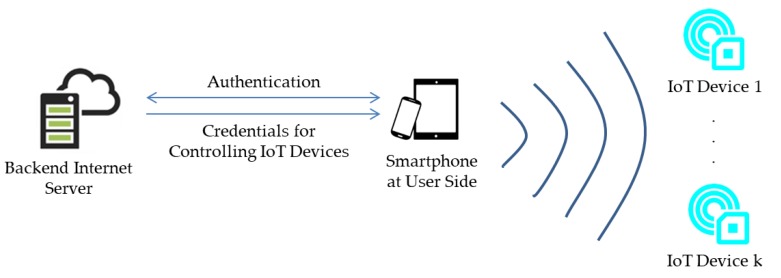
Generalized model of BLE-based applications associated with IoT devices.

This section provides generalized requirements for BLE-based applications associated with IoT devices. As depicted in Figure 3, users use their smartphones to send requests to an Internet server to verify their identities. The server then sends credentials to the user smartphones to enable the smartphones to connect to nearby IoT devices and to control those devices. In that case, the following requirements should be achieved:

- The Internet server must ensure that credentials can only be used by the assigned smartphone.
- An IoT device must be controlled by one smartphone at a time.
- Smartphone users can discover the credentials to control IoT devices which have been used by others even though the IoT devices have no Internet connection abilities.

Table 1 lists possible schemes for IoT devices to identify smartphones. First, after verifying the identities of smartphone users, the Internet server can generate credentials based on the identification information of smartphones [20]. Therefore, an IoT device can restrict access so only the smartphone with the specified identification information can use associated credentials to connect to the device. Similarly, smartphone applications can use smartphone identification information to encrypt credentials for IoT device communication. Then, smartphone applications need to use local identification information to obtain the credentials. If people copy the encrypted credentials to other smartphones, the smartphone applications cannot obtain credentials with incorrect identification information. One of the most critical deficiencies of the above schemes is that smartphone applications may have trouble obtaining the real identification information of the located smartphones.

Malicious users may obtain identification information and associated credentials if smartphone owners jailbreak their smartphones and accidentally install malware on the smartphones. Then, the malicious users can adopt faked identification information to access IoT devices. To address the issue, smartphone users may store credentials in the security elements (SEs) of their smartphones and only perform operations regarding credentials in the SEs. Obviously, the biggest limitation of the scheme is that not every smartphone has built-in SEs. Moreover, when users wish to communicate with IoT devices, the users can request remote servers to send one-time-passwords (OTPs) to user smartphones via SMS messages. However, users may not be able to receive SMSs if their smartphones do not install SIM cards. Also, users may need to pay extra costs to receive SMS messages. Even when the servers send OTPs to users through e-mails rather than SMS messages, the IoT devices need to synchronize with the servers.

Finally, after servers authenticate the users, the servers can generate pseudo-identities for the users and send the pseudo-identities along with associated credentials to users. The smartphone applications can request the users to input PINs and use the PINs to encrypt the data. Then, users can input PINs into smartphone applications to obtain the original pseudo-identities and credentials to communicate with IoT devices. In this case, if malicious users obtain data encrypted with the PINs of others, the malicious users cannot obtain correct pseudo-identities along with associated credentials to connect to IoT devices. One major challenge of the scheme is to deal with the situations that users forget their PINs. Users may further store encrypted PINs to remote servers to address the issue.

## 4. A Privacy-Aware Access-Control Mechanism for BLE-Based Smart Objects

In this section, we present a privacy-aware access-control scheme for enabling BLE-based IoT devices. Let the notation $E/E_{p}$ denotes an elliptic curve E over a prime finite field $E_{p}$, defined by an equation: $y^{2}=x^{3}+ax+b$, where $a,\;b\in F_{p}$ are constants such that $\Delta =4a^{3}+27b^{2}\neq 0$. All points $P_{i}=\left(x_{i},y_{i}\right)$ on E and the infinity point O form a cyclic group G under the operation of point addition R=P+Q defined based on the chord-and-tangent rule. In addition, we define t⋅P=P+P+…+P (t times) as scalar multiplication, where P is a generator of G with order n.

**Figure 4:**
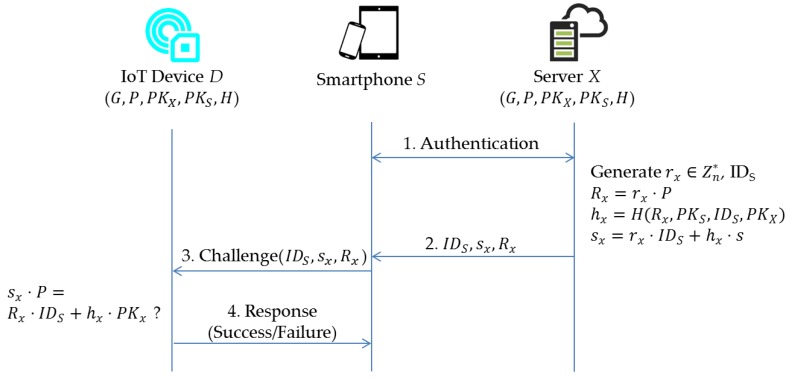
The communication processes of the proposed ECC-based scheme.

- Setup: server *X* generates a group *G* of elliptic curve points with prime order n and determines a generator *P* of *G*. Then, *X* chooses a master key $s\in Z_{n}^{*}$ and a secure hash function $H:{\left\{0,1\right\}}^{*}\times G\to Z_{q}^{*}$. Next, *X* calculates a master public key $PK_{X}=s\cdot P$, and publishes system parameters, i.e., $\left(G,\;P,PK_{X},H\right)$. On the other hand, given params, the smartphone *S* picks a random number $x_{S}\in Z_{n}^{*}$ as it’s own secret value and computes $PK_{S}=x_{S}\cdot P$ as the corresponding public key. During system initialization, a set of system parameters, i.e., $\left(G,\;P,PK_{X},PK_{S},H\right)$, are installed into each IoT device associated with server *X*.
- Verification (Figure 4): first, an authentication between smartphone *S* and server *X* is performed. After authenticating the user, the server generates a pseudo-identity $ID_{S}$ for the user. Given params, s and the identity $ID_{S}$ of smartphone S, server *X* generates a random number $r_{x}\in Z_{n}^{*}$, and calculates $R_{x}=r_{x}\cdot P$, $h_{x}=H\left(R_{x},PK_{S},ID_{S},PK_{X}\right)$ and $s_{x}=r_{x}\cdot ID_{S}+h_{x}\cdot s$ mod *n*. Then, server *X* returns $\left(ID_{S},s_{x},R_{x}\right)$ to the smartphone S which soon forwards it to the device *D*. Then, device *D* checks the validity of $\left(ID_{S},s_{x},R_{x}\right)$ via whether the equation $s_{x}\cdot P=R_{x}\cdot ID_{S}+h_{x}\cdot PK_{x}$ mod *n* holds or not. The correctness of $\left(ID_{S},s_{x},R_{x}\right)$ is presented as follows: $s_{x}\cdot P=\left(r_{x}\cdot ID_{S}+h_{x}\cdot s\right)\cdot P=r_{x}\cdot ID_{S}\cdot P+h_{x}\cdot s\cdot P=R_{x}\cdot ID_{S}+h_{x}\cdot PK_{x}$. If the validity of $\left(ID_{S},s_{x},R_{x}\right)$ holds, device *D* can be operated and accessed by smartphone *S* (and server *X*) and sends a success command as a response to smartphone *S*.

### 4.1. Security Analysis of the Proposed Approach

In this study, we assume that adversary *Adv* models an outside adversary who is able to replace any entity’s public key with specific values chosen by the adversary itself; however, the adversary *Adv* does not know the secret value of Server *X*.

Game 1: The following process is performed between a challenger *C* and an adversary *Adv* during the proposed ECC-based approach between IoT device *D* and server *X*. In Initialization phase, *C* generates s, $x_{S}$ and system parameters, i.e., $\left(G,\;P,PK_{X},PK_{S},H\right)$. Next, *C* sends $\left(G,\;P,PK_{X},PK_{S},H\right)$ to the adversary *Adv*. Next, in Query phase, the adversary *Adv* is able to adaptively issue the following oracle queries, i.e., *RequestPublicKey*(*ID_t_*) and *ReplacePublicKey*(*ID_t_, PK_t_*, *PK_t_^#^*) to *C*, where *t* may be IoT device *D* or server *X*. Finally, in Output phase the adversary *Adv* will output $\left(ID_{t},s_{t},R_{t}\right)$; and, if $true\leftarrow Verify\left(params,ID_{S},s_{x},R_{x}\right)$, the adversary *Adv* wins in Game 1.

- *RequestPublicKey*(*ID_t_*): The oracle takes as input a query (*ID_t_*), where *ID_t_* is the party *t*’s identity. It browses the list *L* and returns the party *t*’s public key *PK_t_*.
- *ReplacePublicKey*(*ID_t_, PK_t_*, *PK_t_^#^*): The oracle takes as input a query (*ID_t_, PK_t_*, *PK_t_^#^*), where *ID_t_* is the party *t*’s identity. This oracle replaces the party *t*’s public key with *PK_t_^#^* and updates the corresponding information in the list *L*.

**Definition** **1.** *The proposed ECC-based* *mechanism between IoT device D and server X is secure against malicious adversaries, if for any polynomial adversary Adv, Succ_j_ is negligible, where Succ_j_ is the success probability that Adv wins in Game 1.*

**Definition** **2.** *(Elliptic Curve Discrete Logarithm Problem; ECDLP). Given a group G of elliptic curve points with prime order n, a generator P of G and a point* x⋅P *, it is computationally infeasible to derive* x *, where* $x\in Z_{n}^{*}$.

**Theorem** **1.** *The proposed ECC-based scheme is existentially secure against malicious adversary in the random oracle model, assuming the hardness of solving ECDLP. If there exist a polynomial-time (pt, q_H_, q_pk_, q_rpk_) adversary* α *which can submit at most q_H_ queries to the oracle Hash(.), q_pk_ queries to the oracle RequestPublicKey(ID_t_) and q_rpk_ queries to the oracle ReplacePublicKey(ID_t_, PK_t_, PK _t_^#^), and* $Succ_{\alpha }$ *is negligible, where* $Succ_{\alpha }$ *is the success probability that* α *wins in game 1, then there exists another algorithm* β *which can solve a random instance of ECDLP in polynomial time with success probability:*

$$Succ_{\beta }\geq \frac{Succ_{\alpha }}{q_{H}\times q_{pk}\times q_{rpk}}$$

**Proof.**  Let α be a polynomial-time adversary that breaks the proposed ECC-based scheme with non-negligible advantage $Succ_{\alpha }$. The goal of this proof is to build a polynomial-time algorithm β which uses α to solve ECDLP. That is, given a random instance (P, Q=x⋅P), it derives the secret x. In Initialization phase, β picks an identity $ID^{*}$ as the challenged identity in game 1, sets $Q=PK_{X}$ and sends public system parameters $\left(G,\;P,PK_{X},PK_{S},H\right)$ to α. In Query phase, α adaptively issue the following oracle queries to β, and each query is unique.

- *Hash* query: For each query, β maintains a list $List_{H}$ storing $<R_{t},PK_{S},ID_{S},PK_{t},h_{t}>$. Upon receiving an *Hash* query for some $<R_{t},PK_{S},ID_{S},PK_{t},\;h_{t}>$ from α, β checks the $List_{H}$ and returns $h_{t}$ to α via the following steps:
  (1) If $<R_{t},PK_{S},ID_{S},PK_{t},\;h_{t}>$ exists in $List_{H}$, β directly returns $h_{t}$ to α.
  (2) Otherwise, it chooses a random value $h_{t}\in Z_{n}^{*}$, adds $<R_{t},PK_{S},ID_{S},PK_{t},\;h_{t}>$ into $List_{H}$, and returns $h_{t}$ to α.
- *RequestPublicKey*(*ID_t_*): Upon receiving a query with an identity *ID_t_* from α, β performs the following steps:
  (1) If $ID_{t}\neq ID^{*}$, β generates three random numbers $a_{t},b_{t},x_{t}\in Z_{n}^{*}$, and performs $R_{t}\cdot ID_{S}\leftarrow a_{t}\cdot P-b_{t}\cdot PK_{t}$, $h_{t}\leftarrow b_{t}$, $s_{t}\leftarrow a_{t}$ and $PK_{t}=x_{t}\cdot P$. Then, β adds $<h_{t},ID_{S},\;R_{t}>$ and $<ID_{S},s_{t},R_{t},PK_{t},x_{t}>$ to the lists $List_{H}$ and $List_{K}$, respectively. Finally, β returns $PK_{t}$ to α.
  (2) Otherwise, β generates three random numbers $a_{t},b_{t},x_{t}\in Z_{n}^{*}$, and sets $ID_{t}\cdot R_{t}\leftarrow a_{t}\cdot P$, $h_{t}\leftarrow b_{t}$, $s_{t}\leftarrow \;⊥$ and $PK_{t}=x_{t}\cdot P$. Then, β adds $<h_{t},ID_{S},R_{t}>$ and $<ID_{S},⊥,\;R_{t},PK_{t},\;x_{t}>$ to the lists $List_{H}$ and $List_{K}$, respectively. Finally, β returns $PK_{t}$ to α.
- *ReplacePublicKey*(*ID_t_*, *PK_t_*, *PK_t_^#^*): Once β receives a query for some (*ID_t_*, *PK_t_*, *PK_t_^#^*) from α, β performs the following steps:
  (1) β looks for $<ID_{S},s_{t},R_{t},PK_{t},x_{t}>$ in the list $List_{K}$. If there exists such a record, β sets *PK_t_* = *PK_t_^#^* and $x_{t}=⊥$.
  (2) Otherwise, β simulate the *RequestPublicKey*(*ID_t_*) query for the identity *ID_t_* and sets *PK_t_*=*PK_t_^#^* and $x_{t}=⊥$.

In the final phase, α successfully outputs $\left(ID_{S},s_{t},R_{t}\right)$ for the target $ID^{*}$ with non-negligible advantage $Succ_{\alpha }$. Based on the forking lemma [21], if we have the polynomial replay of β with the same random tape and different choices of hash oracle, α is able to output another valid signature. Eventually, we will have two valid verification, i.e.,$\;\left(ID_{S},s_{t},R_{t}{}^{\left(j\right)}\right)$, satisfying the following equations $s_{t}{}^{\left(j\right)}=r_{t}\cdot ID_{S}{}^{\left(j\right)}+h_{t}{}^{\left(j\right)}\cdot s$ mod *n*, where *j* = 1 and 2. Now, β can derive the two unknown values $r_{t}$ and s, and outputs s as the solution of the random instance (P, Q=s⋅P) of ECDLP. Next, we analyze β’s success probability $Succ_{\beta }$ of winning in game 1. Note that Event 1 (*E*_1_) denotes that α can forge a valid verification message, i.e., $\left(ID_{S},s_{t},R_{t}\right)$, while Event 2 (*E*_2_) represents that the output $\left(ID_{S},s_{t},R_{t}\right)$ satisfies $ID_{t}=ID^{*}$. It is calculated that $\mathrm{Pr}\left[E_{1}\right]\geq Succ_{\alpha }$, $\mathrm{Pr}\left[E_{2}|E_{1}\right]\geq \frac{1}{q_{H}\times q_{pk}\times q_{rpk}}$ and $Succ_{\beta }=\mathrm{Pr}\left[E_{1}\wedge E_{2}\right]$ = $\mathrm{Pr}\left[E_{1}\right]\mathrm{Pr}\left[E_{2}|E_{1}\right]\geq \frac{Succ_{\alpha }}{q_{H}\times q_{pk}\times q_{rpk}}\;$, where $q_{H}$ is the number of *Hash* query, *q_pk_* is the number of *RequestPublicKey* query and *q_rpk_* is the number of *ReplacePublicKey* query. Hence, the algorithm β can solve ECDLP with, at minimum, the advantage $\;\frac{Succ_{\alpha }}{q_{H}\times q_{pk}\times q_{rpk}}$, where $q_{H}$ denotes the maximum number of queries to *Hash*, *q_pk_* denotes the maximum number of queries to *RequestPublicKey*(*ID_t_*) and *q_rpk_* denotes the maximum number of queries to *ReplacePublicKey*(*ID_t_*, *PK_t_*, *PK_t_^#^*). That contradicts the hardness of solving ECDLP. ☐

### 4.2. Performance Analysis of the Proposed Approach

To evaluate the performance of the proposed approach, we adopt an IoT-based testbed, i.e., Raspberry PI 2, as the implementation platform in the experiment. The Raspberry PI platform is a card-sized single-board computer which offers an ARM GNU/Linux kernel. In general, the Raspberry PI platform is simulated as an IoT-based smart object. The implementation environment is outlined in Table 2. In addition, in the experiment the elliptic curve is with a 384-bit prime n and the random number generator is with 96-bit output sequence, while SHA-3 (512-bit) is used as the one-way hash function. From Table 3, we can see that around 1301 ms is required in terms of the execution time for the required computations at BLE-based IoT devices. Note that in the experiment, the smartphone performs only the job of message forwarding, while the server is responsible for the signature generation. Obviously, both of them will not be performance bottlenecks when performing our proposed privacy-aware access-control scheme. Therefore, we only investigate the performance at the IoT device end in which the signature verification is invoked. 

## 5. Smart Contract-Based Investigation Report Management Framework (SCIRM)

To help application developers develop secure smartphone applications, several countries and organizations such as the European Union Agency for Network and Information Security (ENISA) [23] and the Taiwan Industrial Development Bureau (IDB) [24], have developed guidelines for smartphone application security. However, when users download smartphone applications from marketplaces, users usually cannot know whether the developers of the applications have followed the guidelines to develop the applications. Therefore, organizations such as US National Institute of Standards and Technology (NIST) [25] and the Open Web Application Security Project (OWASP) [26] have developed smartphone application verification guidelines as well as security requirements for smartphone applications. Hence, smartphone application developers can delegate third parties to follow the verification guidelines to check whether their applications satisfy security requirements of the verification guidelines. Furthermore, organization or government agencies may develop certification programs for smartphone applications. For example, the Taiwan IDB has announced the self-regulatory mobile application security certification program [27]. Smartphone application developers can appoint accredited inspection laboratories to inspect whether their applications satisfy applicable security requirements. Consequently, users can decide to only install inspected applications to reduce security risks of using smartphone applications. In light of this, we propose a Smart Contract-based Investigation Report Management (SCIRM) framework for security evaluation of BLE-based applications associated with IoT devices. The framework provides not only ontologies to store verification reports about smartphone applications in a bitcoin-like blockchain, but also standard interfaces based on smart contracts for application verifiers to upload inspection reports to the blockchain. The users are notified once the inspection results of a smartphone application have changed. As a result, the proposed framework can enable users to obtain the latest security information of an application timely. Moreover, users can also obtain historical inspection results on applications by versions, developers, and other application profiles. The proposed framework can also contribute to providing users more information to evaluate application security risks.

**Figure 5:**
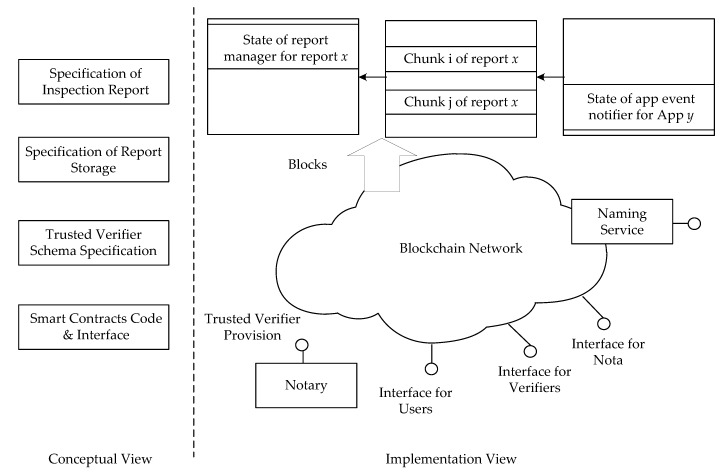
The overview of the proposed SCIRM framework.

Figure 5 provides an overview of the proposed framework. As depicted in Figure 5, the framework contains two views: the conceptual view and the implementation view. The conceptual view is composed of four major standards:

- The Specification of Inspection Report defines the components of an inspection report as well as the format of each component. In general, an inspection report includes a list of inspection results. Each inspection result is for a security objective. An inspector can describe findings about whether an application comply with a security objective in an inspection result.
- The Specification of Report Storage clarifies how to store an inspection report in the blockchain.
- The Trusted Verifier Schema Specification provides properties required to describe a trusted verifier. In general, users may not know the trustworthy of an inspector and the inspection reports it issued. This study assumes that a notary can provide identities and associated public keys for well-known inspectors. For example, Taiwan has the Accreditation Program for Mobile Application Basic Security Evaluation Laboratories [28]. The Taiwan Accreditation Foundation lists information about the accredited laboratories on its Web pages. Therefore, we can simply append blockchain account identities and associated public keys of the laboratories.
- The standard API and the associated smart contract for report management.

The implementation view illustrates the physical components of the framework. We deploy the framework on the Ethereum blockchain system [29]. In the Ethereum blockchain system, people can enclose data in transactions. The blockchain system encapsulates the transactions in blocks. Due to the size limits of a transaction, this study requests a verifier to split an inspection report into several chunks and enclose the chunks into different transactions. The report manager is to manage the inspection reports. When a verifier submits an inspection report to the blockchain, the verifier obtains the compiled byte code of the report manager and creates a new instance of the report manager smart contract. The verifier can then bind the contract to the transactions of the inspection report. As depicted in Figure 5, the Ethereum blockchain system stores the state of each contract in blocks. After a verifier has created a report manager smart contract for an inspection report, people can obtain the state of the contract from a block in the blockchain system and use the state information to find associated transactions storing the chunks of the report. In addition, we define the application event notifier smart contract. A notary can create an application event notifier smart contract for an application. Verifiers can then link the report manager smart contract of the application to the application event notifier smart contract. Therefore, users only need to listen to the application event notifier smart contract to obtain events that an inspection report of the application has been created or obsoleted.

In addition to the constructor, a report manager smart contract provides interfaces for users and verifiers to access information of the associated report. Simply speaking, the report manager smart contract of an inspection report enables users to obtain the contents and status (e.g., active, obsolete, or revoked) of the report. Moreover, report manager smart contracts can trigger events to notify users the status changes of reports. On the other hand, a verifier can update the status of a report and append other reports to the report through the related smart contract. Although users can obtain all inspection reports and related information of smartphone applications by traversing through every block in the blockchain, finding transactions and contract states of a specific smartphone application could be very time-consuming. Therefore, this study proposes to deploy some supernodes for building indexes on transaction attributes and contract states. The supernodes can provide naming services for users to lookup inspection re-ports on a specific smartphone application or reports on applications developed by a specific developer. Finally, in current Ethereum blockchain systems, people only know a transaction or a smart contract is submitted by an identity. Consequently, users cannot determine whether they should trust inspection reports issued by an identity. To address the issue, this study assumes that notaries can provide a list of trusted verifiers as well as information of the verifiers. The notaries may also process complaints of application developers and mark an inspection report as revoked. However, enabling notaries to revoke inspection reports may induce disputes between notaries and verifiers. We leave the report revocation mechanism as our future work.

**Figure 6:**
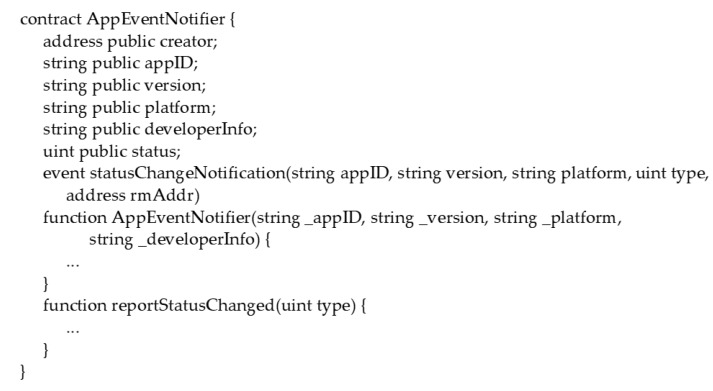
Abstract of the application event notifier smart contract.

Before introducing the report management smart contract, this study will first introduce the application event notifier smart contract. Figure 6 illustrates the abstract interface of an application event notifier smart contract. An application event notifier smart contract contains profiles of a smartphone application. In addition, it provides the *reportStatusChanged* interface for the associated report manger smart contract to inform the application event notifier smart contract that the creator of the report manger has just updated the state of the related inspection report. Consequently, interested people can obtain update events for an inspection report. In this case, SCIRM provides the following types of update events for an inspection report:

SCIRM provides the following types of update events for an inspection report:

- Creation of the inspection report.
- Obsoleting of the inspection report.
- Being extended by another report.

**Figure 7:**
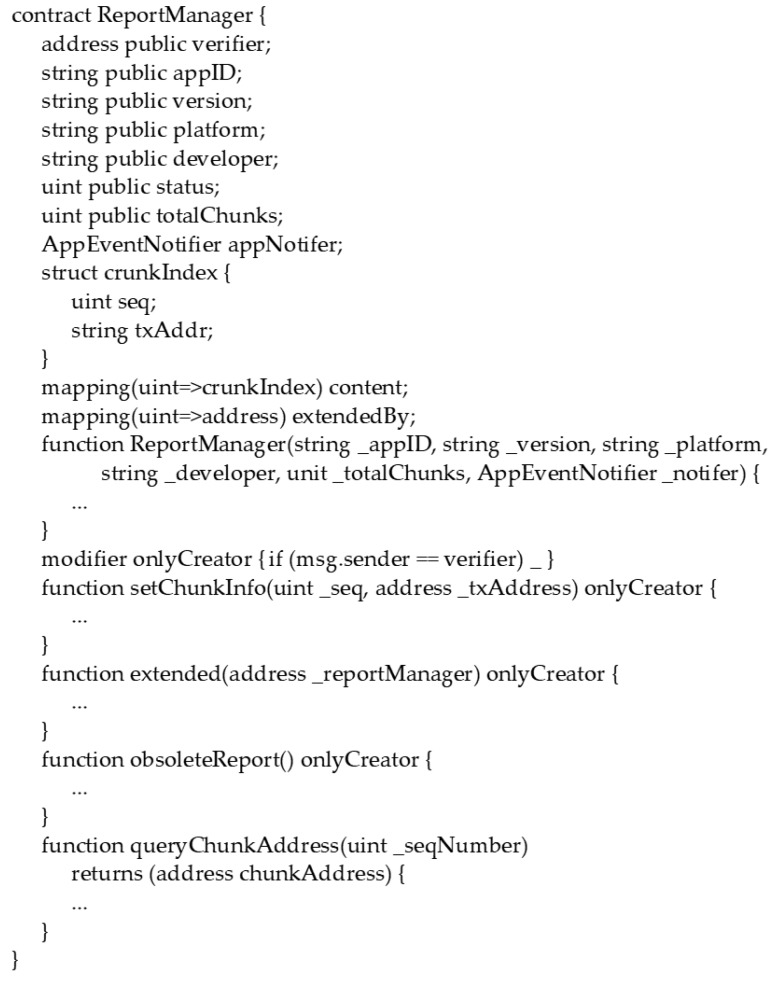
Abstract of the report manager smart contract.

**Figure 8:**
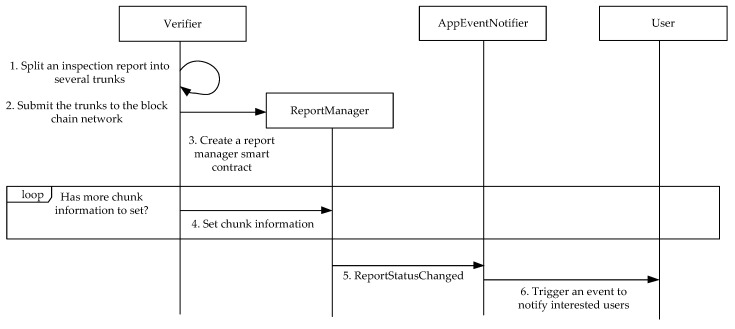
The report creation process.

Note that because contents of transactions are immutable in blockchains, update operations to an inspection report are not provided. The abstract interface of a report manager smart contract is depicted in Figure 7. In addition to the profiles of the associated application, a report manager smart contract contains the status of an inspection report, total chunks of the report, and the addresses of the chunks. As depicted in Figure 8, when a verifier wishes to upload an inspection report for an application, the verifier will first split the report into several chunks and submit transactions containing the chunks to the blockchain network. The verifier then generates a report manager smart contract with application profiles, total chunk number of the report, and the address of the applications event notifier smart contract.

Because the current Ethereum platform only allows using fixed size array as function parameters, the verifier needs to use the *setChunkInfo* function to provide associated transaction addresses one by one. The report manger smart contract will store the addresses in a mapping object. After receiving addresses to access every chunk of the associated report, the report manger smart contract will invoke the *reportStatusChanged* function of related application event notifier. The notifier can then trigger an event to notify interested users of the creation of the report. The verifier can also use the *obsoleteReport* function to obsolete a report, or the *extended* function to represent the situation that the report is extended by another report. The report manger smart contract will also request the associated application event notifier smart contract to notify users when these two functions are called.

**Figure 9:**
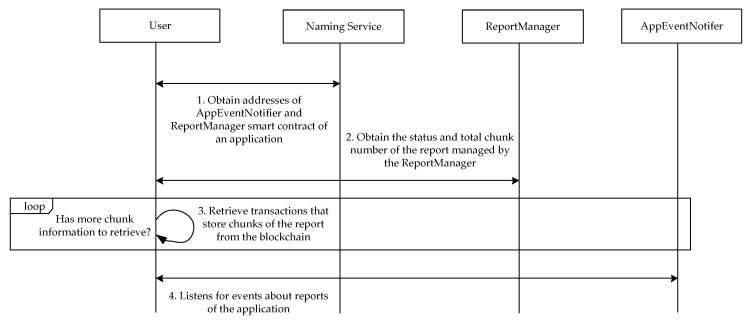
The report query process.

Figure 9 illustrates the process for a user to retrieve inspection reports of an application from a blockchain. When a user downloads a smartphone application from a marketplace, the user can first query the naming service to obtain the address of the *AppEventNotifier* smart contract of the application as well as the addresses of associated *ReportManager* smart contracts. Then, for each *ReportManager* smart contract, the user can obtain the status of the associated inspection report and the verifier to determine whether or not to download the report. If the user decides to download the report, the user can reassemble the report by collecting all the chunks stored in the transactions. Finally, the user can listen to the *AppEventNotifier* smart contract of the application for status changing events of the application inspection reports.

### 5.1. Performance Analysis for SCIRM

**Figure 10:**
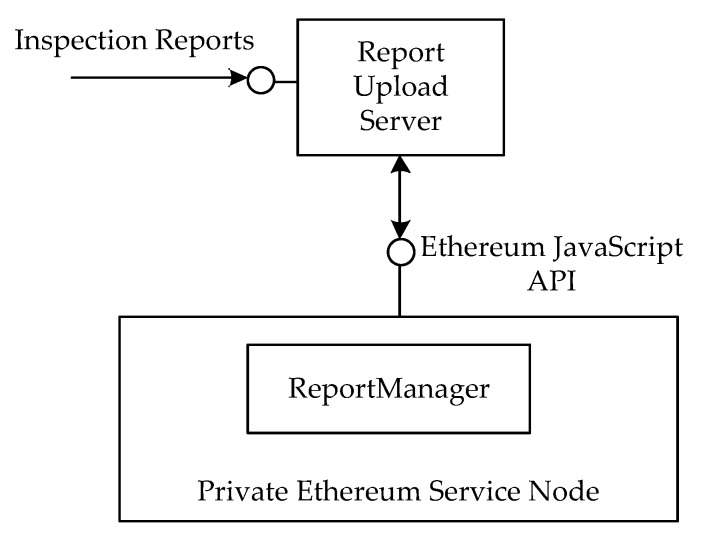
The experimental scenario.

To prove the concept of the proposed framework, we have implemented a prototype system and performed simulation experiment. As shown in Figure 10, we use Node.js to implement a Report Upload Server to handle inspection report uploads. In addition, this study launches a private Ethereum network with a single service node. The report manager communicates with the node via the Ethereum JavaScript API. After receiving an inspection report, the report upload server will split received reports into chucks (Step 1), store the chunks in transactions (Step 2), and send the transaction to the node (Step 3). Next, the report upload server creates a report manager smart contract and sends the addresses to the contract (Step 4).

In the experiment, the report manager and the Ethereum service node are both executed on a desktop equipped with an Intel Core i7-4790 CPU 3.60 GHz CPU and 12 GB RAM running Ubuntu 16.04 LTS. To simplify the experimental environment, this study fixes the *mining difficulty* to 0x4000. Note that the *mining difficulty* is a parameter of the Ethereum and other blockchain platforms. In the blockchain platforms, each participant competes to win the right to generate a data block and store the block in associated blockchains. In this case, a participant tries to find a nonce that the participant can use the nonce to generate a hash value matching specific patterns with the nonce and the data block to be stored. The higher the *mining difficulty*, the more calculating efforts a participant need to perform. In addition, the competition winner can usually receive a specified amount of digital currency as incentives to maintain the platforms continuously. As advances of information technologies, the blockchain platforms usually increase the *mining difficulty* to control the total amount of associated digital currencies. However, increasing the *mining difficulty* may influence the experimental results. Therefore, we fix the *mining difficulty* in our experiment to discuss the differences of using different blocksize. 

An inspection report is about 22 Kbytes (based on the inspection items of the Taiwan self-regulatory mobile application security certification program). This study calculates the average time for the report update server to perform the tasks (from Step 1 to Step 4) in the above paragraph with different maximum chunk size. For each maximum chunk size, this study performs the experiment 20 times. Table 4 summarizes the experimental results in which the experimental result illustrates the time for an inspector to store an inspection report in the blockchain. As listed in the table, the report update server can usually finish the above tasks in reasonable amount of time. On the aspect of report inquiry, a user can usually obtain the inspection report less than a second because the user can just read data in a blockchain directly without any mining behavior.

### 5.2. Security Analysis for SCIRM

The SCIRM framework has the following major security requirements: (a) a malicious person cannot spoof the identity of a verifier to issue an inspection report; (b) unauthorized people cannot tamper with the content of an inspection report; and (c) only the creator of an inspection report can change the status of the report. To fulfill requirement (a), a user can obtain the verifier identity from a report manager smart contract and use the identity to retrieve information of the verifier from the notaries. This study assumes that notaries can check the authenticity of the identities and the related verifier information before the notaries register the data to their databases. Therefore, unless a malicious person can tamper databases of notaries or steal the credential information of a verifier, malicious people cannot impersonate the verifier to issue a report. For the requirement (b), if a malicious person wishes to tamper the content of an inspection report, the person would need to modify the transactions storing the chunks of the report. Moreover, the person may also try to change the variables of the associated report manager smart contract that keep addresses of the transactions to other addresses to link the report manager to a faked report. However, the Ethereum platform has built in security mechanisms to prevent people from tampering existing transactions or contract states in blocks unless the malicious person can control the majority of computing power in the Ethereum network. Therefore, unauthorized person cannot tamper the content of an inspection report while the underlying Ethereum network security mechanism are in place. To meet the requirement (c), this study uses the function modifier of the Ethereum platform to impose constraints so that only the creator of a report manager smart contract can link an inspection report to the smart contract and change the status of the report. Malicious people cannot change the status of the report unless they can obtain the credentials of the creator.

### 5.3. Limitations for SCIRM

We have proposed a SCIRM framework for BLE-based applications to enable smartphone application users to obtain security inspection reports of interested applications using smart contracts. Based on the blockchain technology, users can get historical inspection reports of an application and ensure the integrity of the reports. In addition, application users and other volunteers can collaborate to provide resources needed to host the framework. Therefore, as the framework does not rely on a single party, the framework does not need to consider the business interests of a business company. However, there exist certain limitations that point the way toward future research. First, this study does not address the dispute between application developers and verifiers over contents in inspection reports. In this case, an application developer and a verifier may delegate a mutually agreed third party arbitrator to decide whether or not to invalidate an inspection report or a particular part of a report. Designing and implementing a smart contract to automate the above process would be interesting future work. Second, an application developer may develop an application for different application platforms. Therefore, an application may have different identities in different platforms. To address the issue, we may assign a unique identity to link the same application developed for different platforms. Last but not least, this study currently only provides operations for a whole report. A verifier may wish to invalidate an inspection result item in a report or append a set of new inspection result items to an existing report. However, the finer the granularity, the more complex it is for users to collect inspection result items in different transactions in blockchains. Designing a fine-grained system would be a complicated and interesting problem which is worthy of further studies.

## 6. Conclusions

In this study, we have introduced a robust security paradigm for BLE-based smart objects in the IoT in which two viewpoints on IoT device security and smartphone applications security, respectively, are the focus. First, we addressed the security impact of adopting BLE random address technique on smartphones to prevent malicious adversary from retrieving smartphones’ physical identification codes. Three critical security requirements for designing privacy-aware access-control schemes on BLE-based smart objects are thus derived. Then, a privacy-aware access-control scheme adopting a robust ECC-based crypto-module is presented to fulfill the claimed requirements. Second, we demonstrate the SCIRM framework for efficiently managing security inspection reports for the BLE-based applications on smartphones associated with IoT objects. Based on our analysis, the proposed SCIRM framework is secure and practical for application scenarios in real world.

## Figures and Tables

**Table 1:** List of Schemes to Pair Smartphones to Devices.

Scheme	Deficiencies
Using ID-based keys	Difficulty to obtain identity information. Identity stealing and faking.
Encrypting credentials with identities	
Storing credentials in SE	Not every smartphones have built-in SEs
OTP using out-of-band channels	Extra communication costs. Smartphones may not have SMS capability.
Encrypting credentials and pseudo-identities with user pins	Users may forget their pins

**Table 2:** Implementation Environment.

Environment	Description
IoT Platform	Raspberry PI 2: Broadcom BCM2836 @ 1 GHz Quad-Core ARM Cortex-A7 Architecture, 1 GB DDR2 RAM, SanDisk 16 GB Class 10 SD Card
Programming Language	Eclipse 3.8 with Oracle Java 8
Crypto API	The Bouncy Castle Crypto APIs [22]

**Table 3:** Experiment Results at BLE-based IoT Devices.

Computation Cost	Execution Time
Compute $H(R_{x},PK_{x},ID_{x},PK_{x})$	1.017 ms
Verify $s_{x}\cdot P=R_{x}\cdot ID_{S}+h_{x}\cdot PK_{x}$	0.284 ms

**Table 4:** Experimental results of storing an inspection report in the blockchain.

Maximum Chunk Size (Bytes)	1 K	2 K	4 K	8 K	16 K	32 K
Average time (seconds)	79.6	42.0	24.9	14.0	10.9	7.41
